# Polydopamine Coated CeO_2_ as Radical Scavenger Filler for Aquivion Membranes with High Proton Conductivity

**DOI:** 10.3390/ma14185280

**Published:** 2021-09-14

**Authors:** Roberto D’Amato, Anna Donnadio, Chiara Battocchio, Paola Sassi, Monica Pica, Alessandra Carbone, Irene Gatto, Mario Casciola

**Affiliations:** 1 Department of Chemistry, Biology and Biotechnologies, University of Perugia, via Elce di Sotto 8, 06123 Perugia, Italy; roberto.damato@inl.int (R.D.); paola.sassi@unipg.it (P.S.); mario.casciola@unipg.it (M.C.); 2 Department of Pharmaceutical Sciences, University of Perugia, via del Liceo 1, 06123 Perugia, Italy; monica.pica@unipg.it; 3 CEMIN, Materiali Innovativi Nanostrutturali per Applicazioni Chimica Fisiche e Biomediche, University of Perugia, via Elce di Sotto 8, 06123 Perugia, Italy; 4 Department of Sciences, University of Roma Tre, via della Vasca Navale 79, 00146 Rome, Italy; chiara.battocchio@uniroma3.it; 5 CNR ITAE, via S. Lucia Sopra Contesse, 5, 98126 Messina, Italy; alessandra.carbone@itae.cnr.it (A.C.); irene.gatto@itae.cnr.it (I.G.)

**Keywords:** cerium oxide, polydopamine, radical scavenger, proton conductivity, chemical degradation

## Abstract

CeO_2_ nanoparticles were coated with polydopamine (PDA) by dopamine polymerization in water dispersions of CeO_2_ and characterized by Infrared and Near Edge X-ray Absorption Fine Structure spectroscopy, Transmission Electron Microscopy, Thermogravimetric analysis and X-ray diffraction. The resulting materials (PDAx@CeO_2_, with x = PDA wt% = 10, 25, 50) were employed as fillers of composite proton exchange membranes with Aquivion 830 as ionomer, to reduce the ionomer chemical degradation due to hydroxyl and hydroperoxyl radicals. Membranes, loaded with 3 and 5 wt% PDAx@CeO_2_, were prepared by solution casting and characterized by conductivity measurements at 80 and 110 °C, with relative humidity ranging from 50 to 90%, by accelerated ex situ degradation tests with the Fenton reagent, as well as by in situ open circuit voltage stress tests. In comparison with bare CeO_2_, the PDA coated filler mitigates the conductivity drop occurring at increasing CeO_2_ loading especially at 110 °C and 50% relative humidity but does not alter the radical scavenger efficiency of bare CeO_2_ for loadings up to 4 wt%. Fluoride emission rate data arising from the composite membrane degradation are in agreement with the corresponding changes in membrane mass and conductivity.

## 1. Introduction

In recent years, the need to arrest the effects of climate change is pushing governments worldwide to plan and coordinate efforts to achieve a dramatic reduction in CO_2_ emissions. This requires a revolution in energy supply toward much more flexible renewable energy systems. Hydrogen offers several benefits for simultaneously decarbonizing transport, housing and industrial sectors. Among hydrogen-based technologies, proton exchange membrane (PEM) fuel cells have revealed promising for stationary and automotive applications. Because of the lifetime targets for large-scale stationary applications (≈40,000 h), as well as for automotive applications (>6000 h) [1], the proton exchange membrane durability represents a key element for the longevity of the device. However, several factors limit the membrane’s long-term stability. A source of membrane degradation is due to chemical degradation caused by radical species such as H^●^, OH^●^ and HOO^●^ [2,3,4,5,6], which gives rise to a thinning of the membrane leading to short the lifetime of PEM fuel cells. A strategy to mitigate such radical attacks consists of the incorporation of radical scavengers. For example, the introduction of metal cations, such as Ce^4+^ and Mn^2+^, or their oxides, including CeO_2_ and MnO_2_, revealed to be effective in mitigating the chemical degradation of PFSA (PerFluoroSulfonic Acid) polymers [7,8,9,10,11,12,13,14,15] because, due to the multivalent oxidation state of the metals, they can act as catalysts for the decomposition of hydroxyl and hydroperoxyl radicals.

In a recent paper, it was reported that CeO_2_ nanoparticles dispersed in an Aquivion matrix undergo partial solubilization at relative humidity in the range 50–90%, when the temperature is increased from 80 to 110 °C [16]. As a consequence, for CeO_2_ loadings greater than 2 wt%, a decrease in the composite membrane conductivity was observed with increasing temperature, in such a way that the larger the CeO_2_ loading, the more severe the conductivity drop. It was also found that the formation of a protective shell on the oxide surface, made of fluorophosphonates bonded to cerium ions through the –PO_3_ groups, partially avoided the conductivity drop. However, the organically modified CeO_2_ nanoparticles show reduced radical scavenger activity in comparison with the pristine nanoparticles. A reasonable compromise between stable conductivity and improved membrane stability towards radical was reached by bonding a fluorobenzyl phosphonate (hereafter Bz) to the CeO_2_ surface. Based on these results and taking into account that the phosphonate can be hydrolyzed after long-term operation under conditions of high membrane hydration, it was of interest to coat the oxide surface with a polymeric film that could be hydrolytically more stable than the phosphonate coating.

**Scheme 1:**
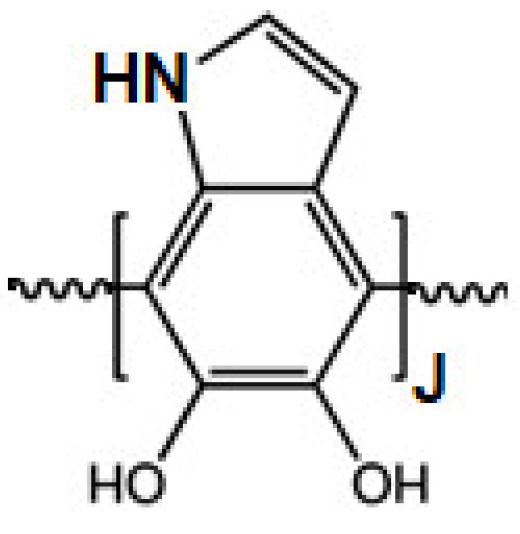
Structure of polydopamine.

To this aim, polydopamine (PDA, Scheme 1) was chosen for its strong and universal adhesion ability and the simple deposition process through self-polymerization in an alkaline aqueous solution [17,18,19,20,21,22,23,24,25,26,27,28,29,30,31,32,33]. 

The preparation of PDA-based materials has rapidly advanced in recent years with a significant expansion in their applications [21,22,23,24,25,26,27,28,29,30,31,32,33,34], becoming one of the most attractive areas within the materials field including surface modification, biosensing [35,36], nanomedicine [37] and systems for energy applications [38,39,40,41,42,43]. In particular, PDA allows obtainment of a beneficial and advantageous interface between CeO_2_ and PFSA improving the lifetime of PEM fuel cells [44].

This paper reports the formation of a PDA film on the surface of CeO_2_ nanoparticles by dopamine polymerization in a water suspension of CeO_2_, and the use of this composite material (PDA@CeO_2_) as a filler of membranes made of Aquivion. 

Membranes containing 3 and 5 wt% filler loadings were characterized by conductivity measurements at 80 and 110 °C, in the RH range 50–90%, to test their stability at increasing temperature. These membranes were also subjected to accelerated ageing by using the Fenton reagent to assess the radical scavenger efficiency of the filler based on the fluoride emission rate (FER). Both conductivity and FER data collected in the present work are compared with the corresponding literature data for composite Aquivion membranes filled with pristine CeO_2_ and Bz@CeO_2_. The most stable membrane was also characterized by Open Circuit Voltage (OCV) stress tests coupled with hydrogen crossover determinations.

## 2. Materials and Methods

### 2.1. Materials

Cerium (III) nitrate hexahydrate (Ce(NO_3_)_3_∙6H_2_O) was from Carlo Erba. A 20 wt% Aquivion dispersion in water (D83-6A, ionomer equivalent weight = 830 g/equiv.) was kindly provided by Solvay Specialty Polymers, Italy. The citric acid (C_6_H_8_O_7_∙H_2_O), dopamine and all other reagents were purchased from Sigma-Aldrich and used without purification.

### 2.2. Synthesis of CeO_2_ and PDA@CeO_2_

Nanopolyhedral CeO_2_ was synthesized by sol-gel followed by thermal decomposition according to the procedure reported in 15. Three composite materials made of cerium dioxide nanoparticles coated with PDA were prepared by reacting, under stirring, a weighed amount of cerium dioxide nanoparticles with 10 mL of a dopamine hydrochloride solution: specifically, 100 mg CeO_2_ with 0.01 M dopamine (hereafter Sample 1), 150 mg CeO_2_ with 0.05 M dopamine (Sample 2) or with 0.1 M dopamine (Sample 3). A suitable amount of NaOH 0.1 M was added to keep pH at 8.5 to achieve a polydopamine film on the nanoparticles. Subsequently, the dispersion was stirred for 24 h at room temperature in dark conditions. The powder was then recovered by centrifugation, washed with water several times and then dried at 80 °C overnight. These composite materials will hereafter be indicated as PDAx@CeO_2_, where x is the PDA weight percentage in the composite. 

### 2.3. Membrane Preparation

The Aquivion dispersion in water was cast on a Petri dish and dried at 80 °C in an oven. The resulting membrane was dissolved in propanol (1 g in 20 mL) at 80 °C. A weighted amount of pristine CeO_2_ or PDA coated CeO_2_ nanoparticles was added to 20 mL of the Aquivion dispersion in propanol. The mixtures were treated with ultrasounds for 10 min, stirred for 2 h, cast by an Elcometer Doctor Blade Film Applicator on a glass support and dried in an oven at 80 °C. After that, all membranes were treated according to the following procedure: 2 h in HCl 1 M, 1 h in H_2_O at room temperature, 2 h at 90 °C and 1 h at 160 °C. Composite membranes, 20–25 μm thick, containing 3% and 5% wt% of PDAx@CeO_2_ were prepared. The same procedure was used to prepare the neat Aquivion membrane.

### 2.4. Ex Situ Accelerated Ageing

Accelerated ex situ ageing tests were performed by treating a membrane sample (ca. 60 mg) with 20 mL of the Fenton reagent (20 ppm iron sulfate, FeSO_4_·7H_2_O, in 30 wt% hydrogen peroxide solution) for 4 h, at 75 °C. The membrane was then washed with deionized water and dried at room temperature. The concentration of fluoride ions in the Fenton’s solution was determined using a Mettler Toledo fluoride ion-selective electrode. The pH was kept in the range of 5–7 using an electrolyte solution (TISAB) with the appropriate total ionic strength adjustment buffer [16].

### 2.5. Conductivity Measurements

The in-plane conductivity was determined according to the four-point impedance technique on 5 cm ± 0.5 cm membrane strips at frequencies ranging from 10 Hz to 100 kHz, with 100 mV, signal amplitude using an Autolab, PGSTAT30 potentiostat/galvanostat equipped with a frequency response analyzer module, as described in ref. [45]. 

### 2.6. Transmission Electron Microscopy

Transmission electron microscopy (TEM) images were collected on powders previously dispersed in ethanol by using a sonicator and then supported and dried on copper grids (200 mesh) coated with Formvar carbon film. A Philips 208 transmission electron microscope, operating at an accelerating voltage of 100 kV, was used. 

### 2.7. X-ray Diffraction

X-ray diffraction (XRD) patterns of powders were collected with a Philips X’Pert PRO MPD diffractometer as described in [46].

### 2.8. Ionic Exchange Capacity Determination

Membrane samples (~250 mg) were dried at 120 °C for 3 h, weighed and then equilibrated in 20 mL of 0.1 M NaCl overnight to exchange the membrane protons with Na^+^ ions. The solution was titrated, in the presence of the membrane, with 0.01 M NaOH through a Radiometer automatic titrimeter (TIM900 TitraLab, Radiometer Copenhagen, Denmark), according to the equilibrium point method. The reported Ionic Exchange Capacity (IEC) values are the average of five replicate titrations [16].

### 2.9. ATR-FTIR

IR spectra were collected by means of Bruker Optics Alpha FTIR instrument equipped with a Platinum-ATR accessory (Bruker Optics, Karlsruhe, Germany). The samples were deposited on the diamond ATR (attenuated total reflection) crystal and their spectrum was recorded at room temperature over the range 5000–400 cm^−1^ with a 2 cm^−1^ resolution.

### 2.10. Near Edge X-ray Absorption Fine Structure (NEXAFS)

NEXAFS spectra were acquired at the ELETTRA storage ring using the BEAR (bending magnet for emission absorption and reflectivity) beamline, installed at the left exit of the 8.1 bending magnet exit. The BEAR beamline has a bending magnet as a source, and beamline optics deliver photons from 5 eV up to 1600 eV; the degree of ellipticity of the beam is selectable. The experimental station is in UHV, and it is equipped with a movable hemispherical electron analyzer and a set of photodiodes to collect angle-resolved photoemission spectra, optical reflectivity and fluorescence yield. In the here reported experiments ammeters to measure drain current from the sample were used. We collected C K-edge and O K-edges spectra at a magic-incidence angle (54.7°) of the linearly polarized photon beam with respect to the sample surface. Both photon energy and spectral resolution were calibrated and experimentally tested using the absorption K-edges of Ar, N_2_ and Ne. The acquired spectra were normalized by subtracting a straight line that fits the part of the spectrum below the edge and imposing an Absorption Intensity value of 1 at 320.00 eV for C K-edge and 560.00 eV for O K-edge. 

### 2.11. In Situ Accelerated Stress Tests

Gas diffusion electrodes (GDEs) were prepared by a spray technique. Sigracet 25- BC Gas Diffusion Layer (SGL), was used as a GDL, and the catalytic ink was deposited onto its surface, as reported elsewhere [47]. The same Pt loading of 0.2 mg cm^−2^ was used for cathode and anode. The GDEs were hot-pressed, at a pressure of 20 kgcm^−2^ for 5 min at 125 °C, onto Aquivion membrane to realize the Membrane-Electrode Assemblies (MEAs). The Accelerated stress tests (AST) in a H_2_/air 25 cm^2^ single cell, at the Open Circuit Voltage and steady-state conditions, were carried out in the following operative conditions: 80 °C, 50% RH, 1.5 bar_abs_, flow rate 1.5 and 2 times the stoichiometry for H_2_ and air, respectively [48]. The tests were performed by connecting the single cell with a commercial test station (Fuel Cell Technologies Inc.), and an AUTOLAB Metrohm Potentiostat/Galvanostat with a 20 A current booster to carry out the electrochemical diagnostics measurements. Linear Sweep Voltammetry (LSV) was carried out by feeding the anode and cathode with hydrogen and nitrogen, respectively, to determine the H_2_ crossover. A potential scan, ranging from 0 to 0.8 V with a scan rate of 4 mVs^−1^, was used to perform the LSV. 

## 3. Results and Discussion

### 3.1. Filler Materials

**Figure 1:**
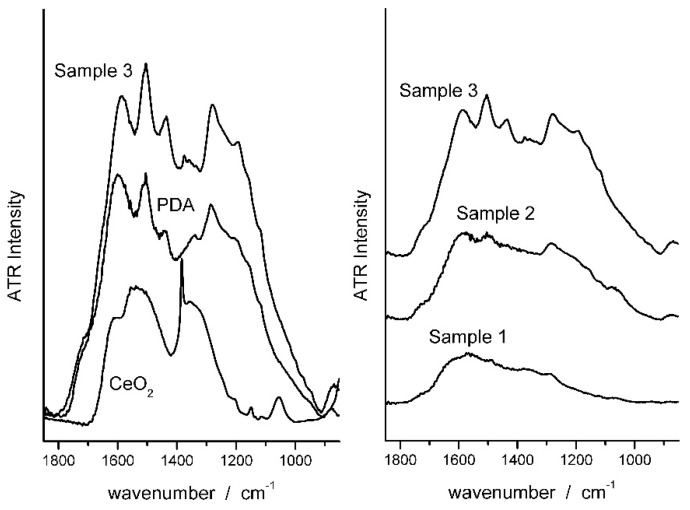
ATR spectra of CeO_2_, PDA and PDAx@ CeO_2_ samples.

The composite samples obtained by reacting dopamine with an aqueous dispersion of CeO_2_ nanoparticles were first characterized by ATR-FTIR spectroscopy to prove the PDA formation. In Figure 1 the ATR spectrum of Sample 3 (see Section 2.2) is compared with the spectra of CeO_2_ and PDA. The main bands of PDA are recognized in the spectrum of the composite. Specifically, the bands centered at 1596 and 1510 cm^−1^ can be attributed to (C=C) and (C–N) stretching modes, respectively, and confirm the presence of aromatic amine species in the coating. The band at ca. 1600 cm^−1^, as well as the feature at 1723 cm^−1^, are assigned to C=O quinone groups. All these peaks increase in intensity as dopamine concentration increases in the reacting mixture, which indicates the increasing PDA concentration in the coatings [49,50,51,52].

**Figure 2:**
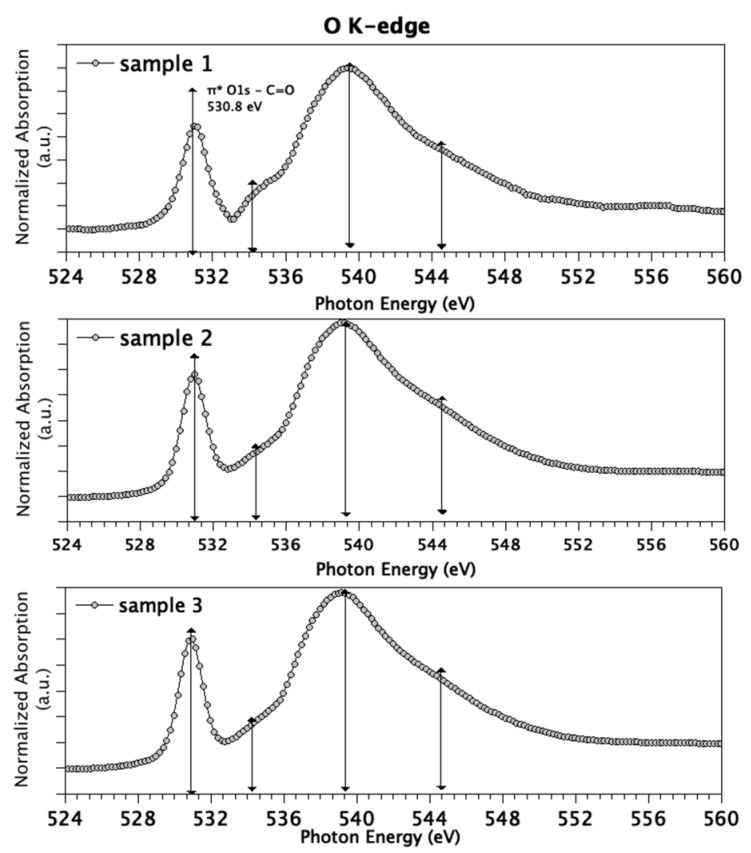
C K-edge spectra measured for Sample 1 (**top**), Sample 2 (**middle**) and Sample 3 (**bottom**).

PDAx@CeO_2_ materials were further characterized by NEXAFS to get information about the PDA oxidation state. All samples show similar features in C K-edge spectra (Figure 2). The main feature appears in the π* region, at about 286 eV, and is attributed to C1s –π*C=O transitions [53]. A couple of features around 289 eV are indicative of the N–containing ring (C=C π* and C–N σ* excitations), confirming the molecular structure integrity. The large and broad feature at about 300 eV in σ* spectral region is associated with C1s – σ*C=O excitations. C K-edge spectra suggest an abundance of C=O functional groups in the examined samples.

**Figure 3:**
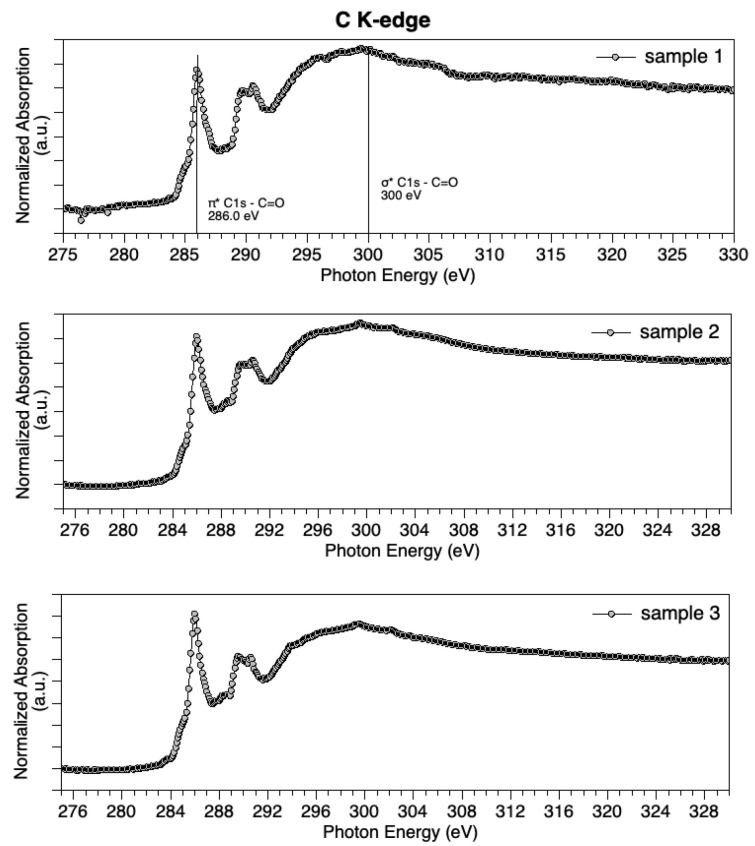
O K-edge spectra measured for Sample 1 (**top**), Sample 2 (**middle**) and Sample 3 (**bottom**).

As with C K-edge spectra, O K-edge spectra (Figure 3) are similar for the three measured samples. The energies of the features in O K-edge spectra and proposed assignments are summarized as follows: the sharp and intense peak centered at 530.8 eV is attributed to the transition of 1 s electrons of C=O groups to antibonding molecular orbitals π*C=O, while the small feature around 534 eV is indicative for transitions of 1 s electrons of hydroxyl-like O atoms to π*O–C and 3 s/σ*O–H [54]. As for the σ* region, features around 540 and 544 eV are associated with O1s C–O and C=O σ* transitions, respectively.

Since in NEXAFS data analysis the so-called *building block approach* can be successfully applied [55] (i.e., the NEXAFS spectrum of a complex molecule or sample can be built by summing up the contribution arising by the different functional groups, weighted for their abundance in the sample), the presence of strong features diagnostic for carbonyl groups, and only weak contributions arising by hydroxyls, suggests that the polymer is mainly in the oxidized state. 

**Figure 4:**
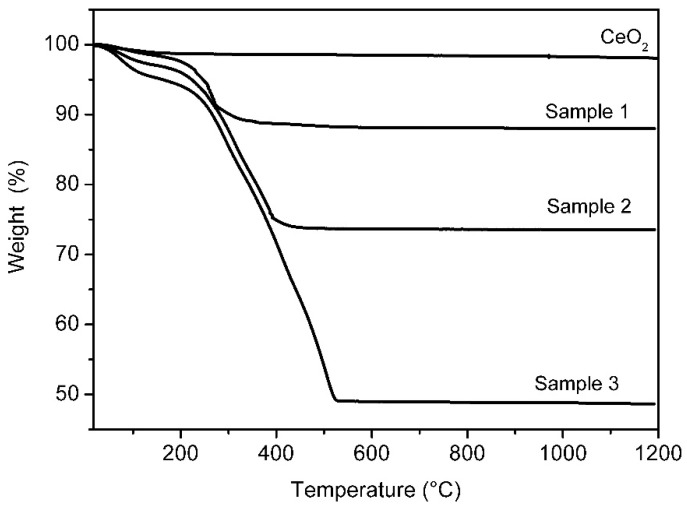
Weight loss curves for bare CeO_2_ and for PDAx@CeO_2_ samples.

The PDA content in the PDAx@CeO_2_ composite samples was determined by thermogravimetric analysis. The weight loss curves for Samples 1, 2 and 3, as well as for bare CeO_2_, are displayed in Figure 4. While CeO_2_ does not present any appreciable loss, the curves of the composites show a small weight loss up to 100 °C, due to the water loss, and a second loss above 200 °C arising from PDA decomposition, which increases with increasing the dopamine concentration used for the polymerization reaction. Based on the second weight loss, the PDA content in anhydrous PDAx@CeO_2_ turned out to be 10.2 wt% (Sample 1), 24.7 wt% (Sample 2) and 49.5 wt% (Sample 3); these samples will be hereafter indicated as PDA10@CeO_2_, PDA25@CeO_2_ and PDA50@CeO_2_, respectively.

**Figure 5:**
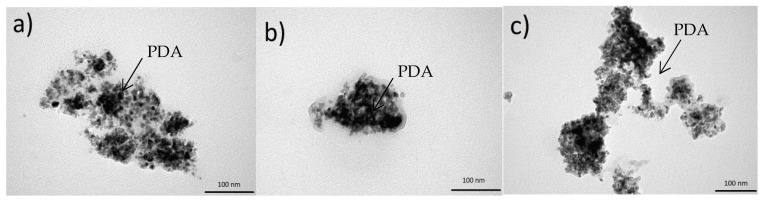
TEM images of (**a**) PDA10@CeO_2_, (**b**) PDA25@CeO_2_ and (**c**) PDA50@CeO_2_.

The morphology of CeO_2_ and the PDAx@CeO_2_ composites was investigated by TEM. The pictures of Figure 5 reveal that PDA can coat the cerium oxide surface forming an irregular layer without affecting the shape and the dimension of the pristine CeO_2_ particles, which in all cases lies around 10 nm. In particular, as the amount of PDA in the composite increases, the thickness of the coating becomes more evident reaching a thickness of some nanometers for the highest PDA content.

To use the PDAx@CeO_2_ materials as fillers of Aquivion composite membranes, we checked that the PDA coating is not soluble in the solvent (propanol) used for membrane preparation. To this aim, 0.05 g of PDA was dispersed in 20 mL of propanol and the mixture was kept under stirring at room temperature for 2 h and then at 80 °C in a closed bottle for 2 h. After centrifugation, the solid was dried at 80 °C. The weight loss curve of the starting material (PDA13@CeO_2_) is coincident, within the experimental error, with the curve of the treated material (PDA13@CeO_2_ PrOH 80) thus indicating that PDA is not soluble under the conditions of membrane preparation (data not shown).

**Figure 6:**
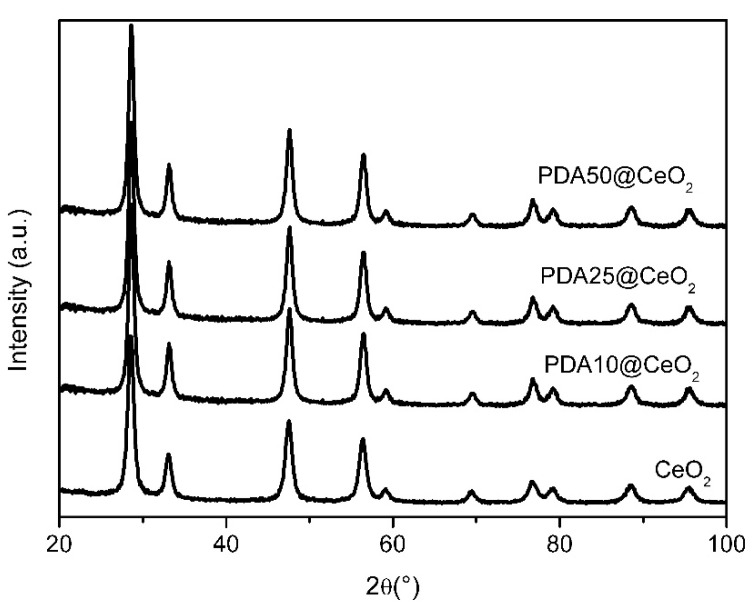
XRD patterns for pristine and coated CeO_2_.

X-ray diffraction (XRD) patterns were collected to reveal possible structural modifications or changes in crystallinity induced by the PDA formation. Figure 6 shows that the position and the intensity of the peaks of bare CeO_2_ do not change in the PDA coated samples suggesting that the presence of the PDA coating does not affect the CeO_2_ crystal structure.

Moreover, in agreement with the TEM images, the particle size calculated using the Scherrer equation lies in the range from 9.9 to 11.2 nm.

The PDA coating of CeO_2_ turned out to be insoluble in propanol at 80 °C, which is the solvent for Aquivion 830: this allowed the PDAx@CeO_2_ materials to be used as fillers of Aquivion based composite membranes.

### 3.2. Composite Membranes

The IEC values (in milliequivalents per gram) of the composite membranes with 3 and 5 wt% PDAx@CeO_2_ loadings (Table 1) are at most by 4% lower than those calculated based on the ionomer weight percentage. In principle, this could be due to the protonation of nitrogen atoms of PDA and/or of surface oxide ions of CeO_2_. The fact that, for the same PDAx@CeO_2_ loading, the IECs show the sequence:

IEC(PDA50) > IEC(PDA25) > IEC(PDA10)

indicates that the protonation of the oxide ions is mainly responsible for the IEC decrease because the amount of cerium oxide in the filler is minimum for PDA50 and maximum for PDA10.

**Figure 7:**
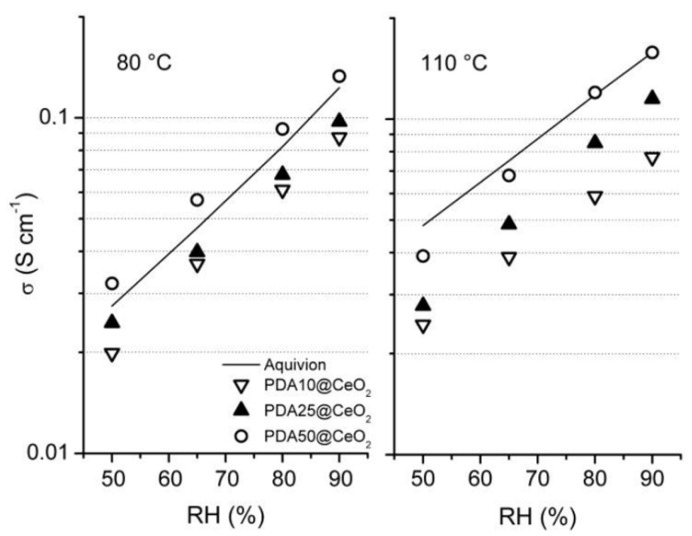
Conductivity as a function of relative humidity, at 80 and 110 °C, for composite Aquivion membranes containing 5 wt% PDAx@CeO_2_. The conductivity of bare Aquivion is also reported.

The conductivity (σ) of membranes containing 3 and 5 wt% PDAx@CeO_2_ (with x = 10, 25 or 50), as well as the conductivity of a membrane containing 6 wt% PDA10@CeO_2_, was determined for RH increasing in the range 50–90%, first at 80 °C and then at 110 °C. In all cases, the plot of logσ as a function of RH is linear. As an example, Figure 7 displays the conductivity of composite membranes containing 5 wt% filler together with that of bare Aquivion. At both temperature and for each RH value, the following conductivity sequence is observed:

σ(PDA50) > σ(PDA25) > σ(PDA10)

which indicates that the conductivity increases with decreasing the CeO_2_ content in the filler. Moreover, going from 80 to 110 °C, the conductivity evolution depends also on the CeO_2_ mass fraction in the filler in such a way that it increases in the presence of PDA50@CeO_2_ but keeps nearly constant in the presence of PDA10@CeO_2_.

A similar trend was already reported for Aquivion/CeO_2_ composite membranes in a recent work [16] where it was shown that the increase in temperature favors the acid-base reaction between cerium oxide and ionomer protons thus causing an IEC decrease which, depending on CeO_2_ content, offsets the expected increase in conductivity. 

**Figure 8:**
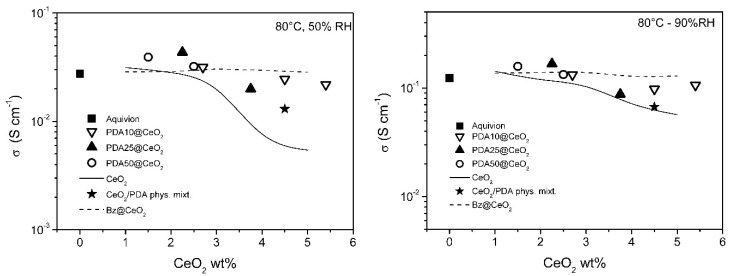
Conductivity as a function of CeO_2_ loading, at 80 °C, for composite Aquivion membranes filled with PDAx@CeO_2_ (x = 10, 25 and 50), as well as with the physical mixture PDA/CeO_2_ (see text). The conductivity of composite Aquivion membranes containing bare CeO_2_ and Bz@CeO_2_ (redrawn from ref. [16]) is reported for comparison.

**Figure 9:**
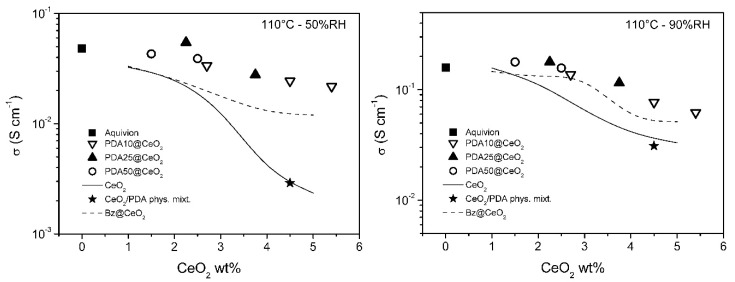
Conductivity as a function of CeO_2_ loading, at 110 °C, for composite Aquivion membranes filled with PDAx@CeO_2_ (x = 10, 25 and 50), as well as with the physical mixture PDA/CeO_2_ (see text). The conductivity of composite Aquivion membranes containing bare CeO_2_ and Bz@CeO_2_ (redrawn from ref. [16]) is reported for comparison.

To get insight into the dependence of the conductivity on CeO_2_ loading, the conductivity of the PDAx@CeO_2_ composite membranes is plotted in Figure 8 and Figure 9 as a function of CeO_2_ wt% in the membrane at constant temperature (80 and 110 °C) and RH (50 and 90%). For comparison, the conductivity of composite Aquivion membranes filled with bare CeO_2_ and Bz@CeO_2_ is also reported [16]. At 80 °C, the conductivity of the composite membranes filled with PDAx@CeO_2_ is weakly dependent on CeO_2_ loading and is close to the conductivity of Aquivion. On the other hand, at 110 °C, the composite membranes become progressively less conductive with increasing of the CeO_2_ loading so that the membrane with 5.4 wt% CeO_2_ is by a factor of about 2.5 less conductive than Aquivion both at 50 and 90% RH.

The CeO_2_ loading being the same, the conductivity of the PDAx@CeO_2_ membranes is similar to the conductivity of the Bz@CeO_2_ membranes except for 110 °C and 50% RH, where the PDAx@CeO_2_ membranes are more conductive by a factor of ~2 at the highest CeO_2_ loadings.

Moreover, the PDAx@CeO_2_ membranes are always more conductive than the corresponding membranes filled with bare CeO_2_ and the difference in conductivity increases with decreasing RH and with increasing filler loading and temperature. As a consequence, at 110 °C and 50% RH, the conductivity of the membrane with PDA10@CeO_2_ containing 4.5 wt% CeO_2_ is by one order of magnitude higher than that of the corresponding membrane containing bare CeO_2_.

It was of interest to prove that the better conductivity of the PDAx@CeO_2_ membranes, in comparison with the corresponding membranes loaded with bare CeO_2_, is indeed due to the presence of the PDA shell on the CeO_2_ surface. To this end, a composite Aquivion membrane containing the same amount of PDA and CeO_2_ as the membrane loaded with 5 wt% PDA10@CeO_2_ (i.e., 0.5 wt% PDA and 4.5 wt% CeO_2_) was prepared by mixing the Aquivion dispersion with a physical mixture of PDA and bare CeO_2_. The conductivity of this membrane, determined at 50 and 90% RH, first at 80 °C and then at 110 °C (the asterisk in Figure 8 and Figure 9), was always lower than the conductivity of the membrane loaded with 5 wt% PDA10@CeO_2_, being in three cases even coincident with the conductivity of the membrane containing 4.5 wt% bare CeO_2_. These results show that it is the PDA coating that efficiently protects the cerium oxide particles against the acidic sulfonic groups of the ionomer, thus avoiding to a large extent the severe conductivity drop occurring with bare CeO_2_.

**Figure 10:**
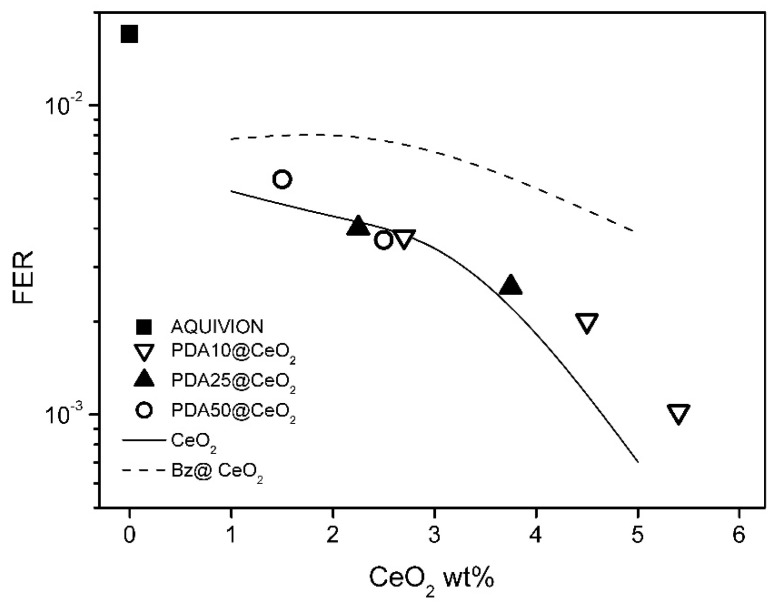
Fluoride emission rate (FER) as a function of CeO_2_ content for composite Aquivion membranes filled with PDA10@CeO_2_, PDA25@CeO_2_ and PDA50@CeO_2_. The FER of composite Aquivion membranes containing bare CeO_2_ and Bz@CeO_2_ (redrawn from ref. [16]) is reported for comparison.

To evaluate the membrane resistance towards radical species generated by the decomposition of hydrogen peroxide, ex situ degradation tests were performed by treating the composite membranes with the Fenton solution (see Experimental section). The results of these tests are expressed in terms of fluoride emission rate, FER, defined as the ratio between the mass of released fluoride ions and the initial mass of the anhydrous membranes. Figure 10 shows the FER values of the PDAx@CeO_2_ composite membranes as a function of CeO_2_ loading and, for comparison, the FER values of composite Aquivion membranes filled with bare CeO_2_ and Bz@CeO_2_. The FER values obtained with PDAx@CeO_2_ are significantly lower than those of Bz@CeO_2_. Like Bz, the PDA coating prevents to a large extent the decrease in conductivity, but, unlike Bz, it does not compromise the radical scavenger activity of CeO_2_. The radical scavenger efficiency of PDAx@CeO_2_ is nearly coincident with that of membranes loaded with bare CeO_2_, for CeO_2_ percentage up to 4 wt%, and similar to that for higher loadings: a FER value of 10^−3^ is indeed obtained with 5.4 wt% of PDA coated CeO_2_ and with 4.7 wt% of bare CeO_2_. It can also be observed that the membranes loaded with 5 wt% PDA50@CeO_2_ and with 3 wt% PDA10@CeO_2_ have the same FER and close CeO_2_ content (2.5 and 2.7 wt%, respectively) but very different PDA content (2.5 and 0.3 wt%, respectively). Thus, the radical scavenger properties of PDAx@CeO_2_ are mainly dependent on the CeO_2_ weight percentage, while the PDA coating does not shield significantly the radical scavenger activity of CeO_2_.

After the Fenton test, the membranes with 3 wt% PDAx@CeO_2_ and the membrane with 6 wt% PDA10@CeO_2_ were washed with 1 M HCl and water, dried at 120 °C and weighed. The percentage weight loss concerning the initial weight of the anhydrous membrane (Table 2) decreases with increasing the CeO_2_ loading in the composite membrane, going from 25.4% for the membrane with 3 wt% PDA50@CeO_2_ to 5.9% for the membrane with 6 wt% PDA10@CeO_2_. 

The conductivity of the aged membranes was also determined at 80 °C and 90% RH (Table 2). The percentage decrease in conductivity with respect to the initial membrane conductivity reflects qualitatively the trend of the weight changes. Thus, both the weight and conductivity of the aged membranes are consistent with the FER data.

**Figure 11:**
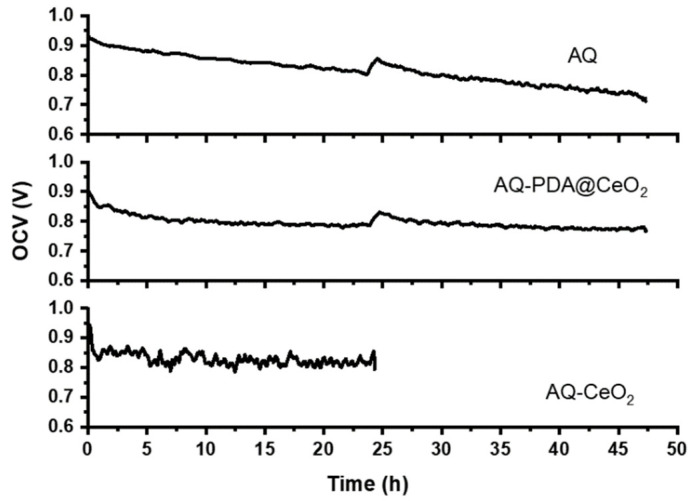
OCV vs. time curves for AQ, AQ-PDA@CeO_2_ and AQ-CeO_2_ membranes.

Based on the results of ex situ characterization, the membrane loaded with 6 wt% PDA10@CeO_2_ (hereafter AQ-PDA@CeO_2_) was selected for MEA realization and in situ characterized by OCV stress tests. Hydrogen crossover measurements were carried out before and during the stress tests after 24 and 47 h from the beginning to check the stability of the membrane. Figure 11 shows the OCV vs. time curves for the bare Aquivion membrane (AQ) and a composite membrane containing 5 wt% CeO_2_ (AQ-CeO_2_). In all cases, after a non-linear drop during the first 3 h, the cell potential decays linearly as a function of time. The same trend is also observed in the time interval between hours 24 and 47 for AQ-PDA@CeO_2_ and AQ after the second hydrogen crossover determination (AQ-CeO_2_ stopped working). 

The OCV decay rate in the linear regions (Table 3) is lower for AQ-PDA@CeO_2_ than for AQ both in the first and in the second time interval of the stress test; as a consequence, the overall OCV decay is ~15% for AQ-PDA@CeO_2_ and ~25% for AQ. Moreover, during the first interval, the decay rate of AQ-PDA@CeO_2_ and AQ@CeO_2_ is similar, thus confirming that the PDA coating does not compromise the radical scavenger activity of CeO_2_.

Consistently with the evolution of the OCV decay rate, the increase in the hydrogen crossover during the stress test (Table 4) is much larger for AQ (~23 times) than for AQ-PDA@CeO_2_ (~2 times).

## 4. Conclusions

Polymerization of dopamine in water suspensions of CeO_2_ allowed preparation of three types of PDA coated CeO_2_ nanoparticles containing 10, 25 and 50 wt% PDA. These materials were used as fillers of Aquivion membranes to improve their chemical stability towards hydroxyl and hydroperoxyl radicals while hindering as much as possible the reaction between the oxide surface and the sulfonic groups of the ionomer.

Composite membranes, loaded with 3 and 5 wt% of each filler, were characterized by conductivity measurements and accelerated degradation tests (based on the Fenton reaction) to assess the radical scavenger efficiency of the filler, and the results of these investigations were compared with those obtained, under the same conditions, for Aquivion membranes containing bare CeO_2_ or Bz@ CeO_2_. It was found that the decrease in conductivity associated with the increase in the CeO_2_ loading is appreciably less severe for membranes containing PDAx@CeO_2_ than for those containing bare CeO_2_, especially at 110 °C and 50% RH. Moreover, the FER of membranes filled with PDAx@CeO_2_ is significantly lower than that of membranes containing Bz@CeO_2_ and, for CeO_2_ percentage up to 4 wt%, it is coincident with that of membranes loaded with bare CeO_2_. Therefore, the PDA coating prevents to a large extent the decrease in conductivity without compromising the radical scavenger activity of CeO_2_. Interestingly, the radical scavenger efficiency of PDAx@CeO_2_ is nearly unaffected by the PDA content as also proved by the results of the OCV stress tests.

## Figures and Tables

**Table 1:** IEC values (meq g^−1^) of PDAx@CeO_2_ composite membranes. The IEC values calculated based on the Aquivion wt% (AQ_calc_) are also reported.

wt%	PDA10	PDA25	PDA50	AQ_calc_
3	1.13	1.15	1.16	1.16
5	1.10	1.12	1.15	1.14

Standard deviation = 0.0032.

**Table 2:** Dry weight and conductivity percentage changes (%∆w and %∆σ) for bare Aquivion and Aquivion composite membranes loaded with PDAx@CeO_2_ after the Fenton test.

Filler	Filler wt%	CeO_2_ wt%	%∆w	%∆σ
Aquivion	0	0	−22	−25
PDA50@CeO_2_	3	1.5	−25	−25
PDA25@CeO_2_	3	2.3	−14	−22
PDA10@CeO_2_	3	2.7	−12	−8
PDA10@CeO_2_	6	5.4	−6	−1

**Table 3:** OCV decay rate for the indicated membranes at 80 °C and 50% RH.

Membrane	OCV Decay Rate (mV h^−1^)		Overall OCV Percentage Loss
	Hour 4–24	Hour 28–47	
AQ-PDA@CeO_2_	1.6	1.5	14.7
AQ-CeO_2_	1.6		15.8
AQ	4.0	4.2	24.6

**Table 4:** Hydrogen crossover for the indicated membranes at 80 °C and 50% RH.

Membrane	H_2_ Crossover at 400 mV (mA cm^−2^)		
	Beginning	24 h	47 h
AQ-PDA@CeO_2_	5.5	12	129
AQ-CeO_2_	1.4	2.3	
AQ	3.4	5.4	6.6

## Data Availability

The data underlying this article will be shared on reasonable request from the corresponding author.
